# S169. MICROGLIAL ACTIVATION AND MORPHOLOGICAL BRAIN ALTERATIONS IN PSYCHOSIS AND PSYCHOSIS RISK

**DOI:** 10.1093/schbul/sby018.956

**Published:** 2018-04-01

**Authors:** Sina Hafizi, Elisa Guma, Alex Koppel, Tania Da Silva, Michael Kiang, Sylvain Houle, Alan Wilson, Pablo Rusjan, Mallar Chakravarty, Romina Mizrahi

**Affiliations:** 1 Centre for Addiction and Mental Health, University of Toronto; 2 Douglas Mental Health University Institute; 3 CAMH; 4 McGill University, Douglas Mental Health University Institute

## Abstract

**Background:**

Abnormal brain structural alterations and microglial activation are implicated in the pathophysiology of psychosis. However, the connection between these two pathologies is not yet well understood. Although previous studies suggested a link between the level of proinflammatory cytokines and abnormalities in brain structure in patients with schizophrenia, there is no in-vivo study investigating whether microglial activation is also linked to morphological brain alterations previously reported in individuals with psychosis and psychosis risk.

**Methods:**

In order to address the current gap in the literature, we investigated microglial activation and structural brain abnormalities in key brain regions affected in psychosis (i.e. hippocampus and dorsolateral prefrontal cortex) of a large group of participants (N = 90) including 35 individuals at clinical high risk (CHR) for psychosis, 27 first-episode psychosis (mostly antipsychotic naïve) patients, and 28 healthy volunteers. All the participants underwent a [18F]FEPPA positron emission tomography (PET) targeting mitochondrial 18 kDa translocator protein (TSPO) to determine microglial activation and a T1 MRI scan to study structural brain characteristics including brain volume, cortical thickness, and hippocampal shape.

**Results:**

Using a vertex-wise analysis, we observed a significant microglial activation-by-diagnostic group interaction in morphological measures across the left hippocampus. We observed associations between microglial activation and outward and inward morphological alterations in the dorsal and ventro-medial portions of the left hippocampus, respectively. These associations were only observed in first-episode psychosis group. There was no association between [18F]FEPPA binding and other structural brain characteristics.

**Discussion:**

Our results, for the first time, suggest a connection between microglial activation and morphological alterations in hippocampus of first-episode psychosis.

